# LIM and SH3 protein 1 (LASP1) differentiates malignant chordomas from less malignant chondrosarcomas

**DOI:** 10.1007/s11060-022-04012-9

**Published:** 2022-05-04

**Authors:** Cas Vanderheijden, Thomas Vaessen, Youssef Yakkioui, Robert Riedl, Yasin Temel, Koos Hovinga, Govert Hoogland

**Affiliations:** 1 Department of Neurosurgery, Maastricht University Medical Center, PO Box 5800, 6202 AZ Maastricht, The Netherlands; 2 School for Mental Health and Neuroscience, Maastricht University, Maastricht, The Netherlands; 3 Department of Pathology, Maastricht University Medical Center, Maastricht, The Netherlands; 4 Department of Neurosurgery, Noordwest Hospital, Alkmaar, The Netherlands; 5 Department of Pathology, Zuyderland Medical Center, Heerlen, The Netherlands

**Keywords:** Chordoma, LASP1, Expression, Tissue microarray, Chondrosarcoma

## Abstract

**Purpose:**

Chordomas are malignant tumors that develop along the neuraxis between skull-base and sacrum. Chondrosarcomas show similarities with chordomas, yet show less malignant behavior. LIM and SH3 protein 1 (LASP1) is a cytoskeletal protein known to promote the malignant behavior of tumors. LASP1 was previously identified as a possibly overexpressed protein in a chordoma proteomics experiment. In this study we compare LASP1 expression in chordoma and chondrosarcoma tissue.

**Methods:**

Biopsies of primary tumors were collected from surgically treated chordoma (n = 6) and chondrosarcoma (n = 6) patients, flash-frozen upon collection and collectively analyzed for LASP1 RNA (real-time PCR) and protein expression (western blotting). Additionally, tissue micro array (TMA)-based immunohistochemistry was applied to an archive of 31 chordoma and 1 chondrosarcoma specimen.

**Results:**

In chordoma samples, LASP1 mRNA was detected in 4/6 cases and a strong 36 kDa immunoreactive protein band was observed in 4/5 cases. In contrast, 0/6 chondrosarcoma samples showed detectable levels of LASP1 mRNA and only a weak 36 kDa band was observed in 4/5 cases. Immunohistochemical analysis showed LASP1 expression in all chordoma samples, whereas chondrosarcoma specimen did not show immunoreactivity.

**Conclusion:**

LASP1 is strongly expressed in the majority of chordoma cases and shows low expression in chondrosarcoma tissue. Since LASP1 is known to function as oncogene and regulate cell proliferation in other tumor types, this study implicates a role for LASP1 in chordoma biology. Further studies are warranted to improve understanding of LASP1’s expression and functioning within chordoma, both in vitro and in vivo.

**Supplementary Information:**

The online version contains supplementary material available at 10.1007/s11060-022-04012-9.

## Introduction

Chordomas are rare tumors thought to develop from remnants of the embryonic notochord. The incidence is 0.8 per 1,000,000 per year and there is a slight male predominance [1, 2]. Although chordomas can occur anywhere along the spinal axis, the skull base and sacrum both account for 1/3rd of cases [1, 3]. These tumors are histologically classified as low-grade neoplasms, but their biological behavior is malignant. Distant metastases are less frequently observed, but patients tend to succumb from progressive local disease [2, 4–6]. Median survival is 7.7 years, but individual prognosis varies considerably [7]. The current mainstay treatments are maximal safe resection and (particle beam) radiotherapy. Due to their invasiveness and both chemo and radioresistance, achieving disease control remains a major challenge and after recurrence, clinical progression is characterized by increased local aggressiveness [2]. As targeted therapies have not yet been able to significantly improve prognosis, it is of paramount importance to increase our understanding of basic chordoma tumor biology.

Based on a chordomas and chondrosarcomas proteomics iTRAQ (isobaric tags for relative and absolute quantitation) experiment [8], LIM and SH3 protein 1 (LASP1) was previously suggested to be involved in chordoma pathobiology. LASP1 has been described to be physiologically expressed at a basal level in tissues and overexpression in cancer is typically associated with worse prognosis [9, 10]. Researchers have reported elevated expression in various cancers, including non-small cell lung cancer [11], esophageal squamous cell carcinoma [12], colorectal carcinoma (CRC) [13], gallbladder carcinoma [14], breast [15] and ovarian [16] tumors, hepatocellular carcinoma (HCC) [17, 18] as well as pancreatic [19] and gastric [20] cancers. LASP1 expression is also reported to be elevated in certain human fetal tissues (including fetal brain, liver and umbilical vein endothelium) and is thought to be involved in cellular migration and differentiation [21, 22]. Furthermore, LASP1 has been shown to be involved in mouse vertebral chondrocyte development [23], which is interesting considering the notochordal fate hypothesis of chordoma [9].

In 1995, LASP1 cDNA was cloned from a cDNA library derived from breast-cancer metastatic lymph node by Tomasetto et al. [24]. The gene was mapped to the long arm of chromosome 17 (17q12-21). The 261-residue protein contains a LIM (Lin-11, Isl-1, Mec-3) domain at its N-terminal. Its C-terminal harbors a Src homology 3 (SH3) domain. In between, two nebulin-like repeats are located which facilitate binding to F-actin filaments [10]. Interestingly, the 17q arm on which the *LASP1* gene resides, is known to be amplified in in some chordomas, showing gains of chromosomal material in 21% of chordomas [25].

Hitherto, LASP1 research has focused on tumors of epithelial origin, and sarcomas have been relatively neglected. LASP1 expression has not been actively investigated in chordomas. In contrast, chondrosarcomas bear similarity to chordomas, both in terms of morphology and localization, although they behave less aggressively. These tumors are thought to originate from mesenchymal tissues, rather than notochordal remnants and they lack the brachyury expression typical of chordomas [26]. Considering LASP1’s established association with malignancy, comparison between chordomas and chondrosarcomas in terms of LASP1 expression might provide further insights into the pathobiological difference between the two. The comparison between the two tumors is valuable as these tumors share many characteristics but chordomas typically behave more aggressively and an explanation for this difference has not yet been established.

This study aims to investigate the expression of LASP1 in chordoma, and aims to gain first insight into its cellular localization by virtue of tissue microarray-based immunohistochemistry. To illustrate LASP1’s presumed role in malignant behavior, expression in chordomas is compared to chondrosarcomas.

## Materials and methods

### Samples

Human skull-base chordomas and chondrosarcomas used in this study were diagnosed at the pathology department in our center according the WHO classification of tumors of soft tissue and bone [27]. Samples were intraoperatively obtained, immediately frozen upon collection and stored at − 80 °C until mRNA and protein analysis. Samples for use in the tissue micro array were collected from our FFPE archive (with specimens collected until March 2017). The storage and use of tissue and patient data were performed in agreement with the "Code for Proper Secondary Use of Human Tissue in the Netherlands" (https://www.coreon.org). Due to the low prevalence and slow growth rate of chordomas, limited tissue was available and tissues were used for the assessment of either RNA expression or protein levels. For chondrosarcoma, five samples that were used for gene expression analysis were also used for western blot analysis. For one sample we only assessed RNA expression due to limited tissue availability. Patient numbers per analysis were: (i) mRNA expression analysis: n = 6 chordoma, n = 6 chondrosarcoma; (ii) protein expression analysis: n = 5 chordoma, n = 5 chondrosarcomas; (iii) tissue microarray immunohistochemistry: n = 31 chordoma, n = 1 chondrosarcoma).

### RNA expression

Tissue samples were lysed with TRIzol® Reagent (1 ml per 50 mg tissue; Invitrogen, Carlsbad, USA) using a Mini-Beadbeater (BioSpec products, Bartlesville, USA), followed by RNA extraction according to manufacturer’s instructions and quantification of RNA by NanoDrop (ThermoFischer Scientific, Waltham, USA). RNA (1000 ng) was reverse transcribed using a RevertAid™ first strand cDNA synthesis kit with Oligo (dT)_18_ primer (ThermoFischer Scientific, Waltham, USA). Real-time quantitative PCR was then performed with the transcribed cDNA, 10 µl SensiMix (SensiMix™ SYBR® & Fluorescein Kit, Bioline, London, UK) and *LASP1* forward- and reverse primer (250 nM each; Sigma-Aldrich, Saint Louis, USA) in a total volume of 20 µl. *LASP1* primer sequences: forward: 5′-CAGCCCCAGTCTCCATACAG-3′ and reverse: 5′-ATACTGATGTCGCGGCGG-3′ [28]. A549 cell line (Sigma-Aldrich) cDNA (obtained by the same method as used for tissue samples) was used as positive control for the *LASP1* gene expression. Beta-actin (*ACTB*) was used as reference gene (*ACTB* forward: 5′-GCACTCTTCCAGCCTTCCTT-3′; reverse: 5′-CGTACAGGTCTTTGCGGATG-3′) [29]. An A549 reverse transcriptase negative sample and a no-template sample were used as negative controls. Reactions were run in a LightCycler 480 system (Roche, Basel, Switzerland), using the following parameters: 10 min pre-incubation at 95 °C, 40 cycles (15 s at 95 °C, 15 s at 60 °C, 15 s at 72 °C). For both primer sets, the real-time PCR primer efficiency was validated by a positive control serial dilution.

### Protein expression

LASP1 protein expression was assessed by western blot in five chordoma samples and five chondrosarcoma samples from different patients. HeLa cell (Sigma-Aldrich) lysates were used as a positive control. Intraoperatively obtained biopsies were flash frozen in liquid nitrogen and stored at − 80 °C until follow-up analysis by western blotting.

For blotting, tumors samples were lysed in TRIzol® Reagent (1 ml per 50 mg tissue) using a Mini-Beadbeater, followed by a protein extraction using according to manufacturer’s instructions. Next, the total protein concentration in each sample was estimated by the bicinchoninic acid assay (ThermoFisher). Proteins (20 µg per sample) were then resolved on a 10% SDS-poly-acrylamide gel (electrophoresis at 100 V for 2 h at room temperature (RT)) using a Mini-PROTEAN® Tetra System (Bio-rad, Hercules, CA, USA) and transferred at 100 V for 1 h on ice to a nitrocellulose membrane. Membranes were subsequently blocked by Odyssey blocking buffer (1 h at room temperature (RT)), incubated simultaneously with primary antibodies polyclonal rabbit anti-LASP1 (HPA012072, Sigma-Aldrich; 1:10,000 diluted in Odyssey blocking buffer) and monoclonal mouse anti-beta actin (sc-81178, Santa-Cruz, Dallas, USA; 1:2,000 diluted in Odyssey blocking buffer) overnight at 4 °C, washed (subsequently in phosphate buffered saline (PBS), PBS-Tween 0.1% and PBS, 5 min each) and then with secondary goat anti-rabbit antibody (IRDye800; LI-COR Biosciences, Lincoln, USA; 1:10,000 diluted in Odyssey blocking buffer) and donkey anti-mouse (IRDye680; LI-COR Biosciences; 1:10,000 diluted in Odyssey blocking buffer) 1 h at RT. Finally, immunoreactive bands were visualized by an Odyssey CLx imaging system and analyzed using Image Studio version 5.2 (LI-COR Biosciences, Lincoln, USA).

### Tissue microarray immunohistochemistry

Intraoperatively obtained biopsies (n = 31 chordoma, n = 1 chondrosarcoma) were fixed overnight in 4% paraformaldehyde and then paraffin embedded. Representative sections of tumor tissue regions were identified on hematoxylin–eosin (HE) stained 4 µm sections by a dedicated neuropathologist (RR). Tissues blocks were needle-punched and 2 mm cores were collectively embedded in a receiver paraffin block. Four µm sections (one for H&E and one for LASP1 staining) were cut and mounted on a positively charged glass slide. Slides were subsequently deparaffinized, rehydrated and incubated with polyclonal rabbit anti-LASP1 [HPA012072, Sigma-Aldrich; 1:10,000 diluted in Tris buffered saline (TBS)] and the EnVision FLEX Mini Kit, High pH (GV82311-2, Dako Omnis, Agilent, Santa Clara, USA) overnight at 4 °C, according to the manufacturer’s instructions. After washing (15 min in TBS, TBS-Triton 0.1%, TBS, each), sections were incubated with HRP-conjugated anti-rabbit antibody, according to manufacturer’s instructions (EnVision FLEX kit, 2 h at RT) and then with 2-diaminobenzidine as the substrate chromogen. Negative control sections were submitted to the same procedure, with the exception that anti-LASP1 was omitted. Hematoxylin–eosin counterstaining was performed to enhance visualization of tissue morphology. Sections were imaged using the Ventana iScan (Roche) at 40× magnification. Evaluation of immunoreactivity was performed by distinguishing distinctly positive from negative cores independently by both CV and TV.

## Results

### RNA expression

*LASP1* expression was detected in A549 cells, used as positive control, at a Ct of 26.03. Both the A549 reverse transcriptase negative sample and the no-template control sample showed no detectable level of *LASP1*. In patient samples, *ACTB* was expressed at mean Ct 24.43 in chordoma and Ct 25.46 in chondrosarcoma. Four chordoma and none of the chondrosarcoma samples showed detectable *LASP1* cDNA levels. The mean Ct of the *LASP1*-positive chordoma samples was 33.1 (range 32.06–34.51).

### Protein expression

**Fig. 1:**
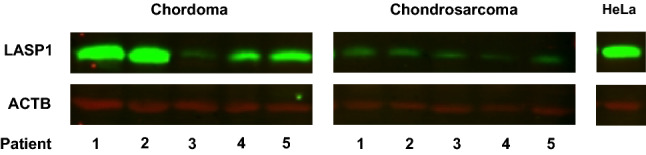
Western blot of skull-base chordoma, chondrosarcoma and HeLa (control) samples showing LASP1 and ACTB protein expression at 36 and 42 kDa, respectively

HeLa cell lysates, used as a positive control, showed a LASP1 immunoreactive band at 36 kDa and reference protein ACTB was consistently detected at 43 kDa in all samples (Fig. 1). Four chordoma samples also showed a prominent LASP1 immunoreactive band at 36 kDa. Though all chondrosarcoma samples also exhibited a LASP1 immunoreactive band at 36 kDa, the expression levels were strongly reduced compared to those in chordoma samples.

### Tissue microarray immunohistochemistry

**Fig. 2:**
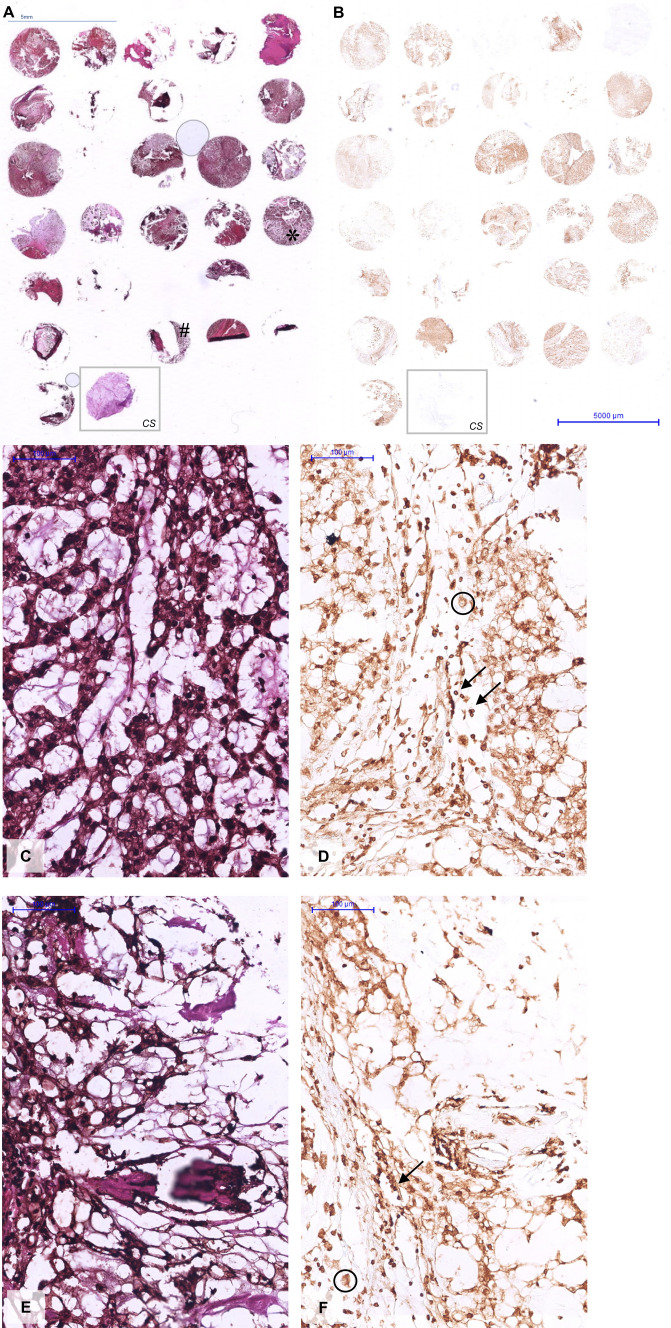
Tissue micro arrays of chordoma cores from 31 patients and a chondrosarcoma core (CS; within the grey rectangle) from 1 patient that were H&E stained (**A**). The adjacent section of the respective patient samples was immunohistochemically stained for LASP1 (**B**), showing a clear expression in most chordoma samples yet no signal in the CS sample. Enlargements of * and # show LASP1 expression in both the nucleus (black arrows; **D**–**F**) and the cytoplasm (black circles; **D**–**F**). Magnification 40X

Immunohistochemical evaluation showed a strong immunoreactive LASP1 signal in 23 chordoma specimen (Fig. 2). Eight specimens could not be assessed due to low cell densities or lack of representative chordoma cell populations (n = 4) or tissue/core loss during staining (n = 4). The remaining 23 chordoma samples, showed prominent LASP1 positivity in a heterogenic fashion. All chordoma samples show areas of strong cytoplasmic and/or nuclear immunoreactivity an also contain areas without said positivity. In all cases, no vacuolar positivity was observed. No areas of poorly differentiated chordoma or convincingly chondroid chordoma areas were observed in the sampled cores. The chondrosarcoma sample did not show LASP1 immunoreactivity (Fig. 2).

## Discussion

Chordoma is a rare neoplastic entity that shows unique biological behavior. Although tumor progression may be relatively slow compared to other cancers, its local invasiveness and frequent recurrence poses a major therapeutic challenge, ultimately resulting in many patients succumbing to progressive local disease. Chondrosarcomas bear many similarities to chordomas, but typically show a more favorable disease course. Thus, differentiating between the two entities is important and it is interesting to study differentially expressed factors such as LASP1, that might be implicated in the tumor biology of chordoma. Our purpose was to evaluate the expression of LASP1 in primary skull-base chordoma and chondrosarcoma and to gain an understanding of its sub-cellular localization.

We observed prominent expression of LASP1 in chordoma. Results from the gene expression experiment, the western blot data and immunohistochemistry in particular show convincingly, for the first time, that LASP1 is expressed in the majority of chordomas. Not all chordomas show LASP1 positivity in the gene expression and western blot assay, thus a degree of heterogeneity (both between tumors, and within different cell populations of the same tumor) may exist to account for this difference. Long-term follow up of patients with chordomas showing LASP1 positivity versus those that do not, may offer valuable insights in disease aggressiveness and a possible role of LASP1. In our experiments chondrosarcomas did not show similar elevated LASP1 expression both in RNA expression and protein expression. In terms of protein localization within primary skull base chordoma cells, the TMA has shown LASP1 to be expressed in both the cytoplasm and the nucleus.

Residing primarily in the cytosol, LASP1 stabilizes filamentous bundles of the cytoskeleton via its two F-actin binding nebulin repeat units [30]. Additionally, LASP1 can interact with numerous other proteins, including but not limited to, zyxin, kelch related protein 1 (Krp1) and dynamin (a complete overview of LASP1 binding partners is beyond the scope of this paper but is reviewed by Butt et al. [21]). Finally, LASP1 has also been shown to localize to membrane extensions [31], such as podosomes/invadopodia, lamellipodia and focal adhesion points [30–33].

Next to a cytosolic location, LASP1 can undergo nuclear translocation as was first observed in the nucleus of human breast carcinoma cells [34]. Nuclear localization of LASP1 has since then also been observed in hepatocellular carcinoma [18], bladder carcinoma [35] and medulloblastoma [36]. Phosphorylation of LASP1 facilitates dissociation from cytoplasmic actin at focal adhesion points. Mihlan et al. [37] showed LASP1 to bind with its SH3 domain to the first proline-rich region of the nuclear shuttle protein zona occludens protein 2 (ZO-2), which can then be transported into the nucleus. Conversely, nuclear export of LASP1 can be achieved by binding of its nuclear export signal to the nuclear export protein CRM1 (a.k.a. Exportin-1), followed by protein phosphatase 2B-induced dephosphorylation. This results in a return of LASP1 to the cytosol and the cell membrane. Thus, LASP1 may shuttle between the membrane and the nucleus, which may explain why our immunohistochemical data show nuclear LASP1 immunoreactivity. To the best of the authors’ knowledge, this nuclear shuttling mechanism of LASP1 has not yet been investigated in chordomas and chondrosarcomas, specifically. The physiological significance of nuclear LASP1 may be explained by its structure. Containing a tandem repeat of two zinc fingers inherent to its LIM domain, LASP1 could in theory alter transcription via binding to DNA [38, 39]. For example, Duval-Noelle et al. [40] have shown that in invasive breast cancer nuclear LASP1 can serve as a hub for several epigenetic proteins such as including Snail1 [41, 42]. They also observed that (nuclear) LASP1 expression positively correlate with the degree of malignancy. In line with this observation, Grunewald et al. [34] showed that nuclear localization and increased cytosolic expression of LASP1 both correlated with the degree of invasiveness in breast cancer.

No immunoreactivity was observed in the sole chondrosarcoma core although this core was not as densely cellular as some of the chordoma cores, which is a limitation of this study. The TMA design with one core per sample carries inherent limitations such as a small amount of tissue per tumor (sampling error) and variations in tissue composition and cell density between samples. This effect is partially mitigated by including 31 different chordomas in the array. Furthermore, only one chondrosarcoma sample was available for immunohistochemistry in this assay and one must be careful to extrapolate. However, both on the mRNA level and in the western blot assay, the differences between chordoma and chondrosarcoma remain prominent.

On the TMA, chondroid chordoma tumors were not seen yet a risk of sampling error is inherent to the TMA design. A further distinction between a (chondroid) chordoma and a chondrosarcoma can be made immunohistochemically, using classical markers such as epithelial membrane antigen (EMA), cytokeratins (CK; CK8, CK18 and CK19) and brachyury [43]. Evaluation of the TMA used here shows that LASP1 differentiates between the chordomas and the sole chondrosarcoma, and western blot and real-time quantitative PCR results do suggest LASP1’s diagnostic potential as well. However, further immunohistochemical research evaluating a larger number of chondrosarcomas is required to assess the specificity and sensitivity of LASP1 in distinguishing (chondroid) chordomas from chondrosarcomas on immunohistochemistry.

Increased LASP1 has been shown in other tumors to correlate with a higher degree of malignant behavior, invasiveness and pro-migratory behavior. The question rises if this holds true for chordoma as well. One chordoma showed no expression of LASP1 on western blot and two did not show detectable levels of mRNA on real-time quantitative PCR. Chordomas behave more aggressively than chondrosarcomas and a distinct difference in LASP1 expression between the two entities becomes evident from the data presented. Assuming a causative relationship between these observations is tempting and future investigations will need to shed more light on this possible pathobiological difference.

The data presented highlight the expression of LASP1 in chordoma, and its relative lack thereof in chondrosarcoma. Chordomas and chondrosarcomas are thought to originate from different cell populations (notochordal versus mesenchymal populations), but carry many morphological and functional similarities. However, it is relevant to study expressional differences that may explain the substantial difference in clinical behavior between the two tumors. LASP1 has already been shown in a multitude of other tumors to increase malignant, invasive and pro-proliferative behavior. Its prominent expression in chordomas rather than chondrosarcomas may explain part of chordomas’ aggressive and invasive growth pattern in vivo. Furthermore, LASP1 might be useful in de diagnostic differentiation between the two tumors, although this should first be confirmed in a larger series.

## Conclusion

LASP1 is a known oncogene that plays a role in the regulation of cytoskeletal activities. LASP1 expression has not been demonstrated before in chordoma. Expression at both mRNA and protein level indicate that LASP1 is prominently expressed in the majority of chordomas, both in cytosolic and nuclear compartments. Chondrosarcomas, known for their more benign biological behavior, lack this strong expression. Based on this observation it is tempting to hypothesize that this oncoprotein is involved in the invasive character of chordomas. This role may be exerted both by their structural and signaling functions in the nucleus and at the cytoskeleton.

## Supplementary Information

Below is the link to the electronic supplementary material.

Supplementary file1 (DOCX 579 KB)
