# Supplementary material for: LIM and SH3 protein 1 (LASP1) differentiates malignant chordomas from less malignant chondrosarcomas
Source: J Neurooncol. 2022 May 4;158(1):81–8. doi: 10.1007/s11060-022-04012-9 (PMC9166821; doi:10.1007/s11060-022-04012-9)
Supplement: Supplementary file 1 — Supplementary file1 (DOCX 579 KB) [file 11060_2022_4012_MOESM1_ESM.docx]

**SUPPLEMENT**

Complete western blot of chordoma (n=5) versus chondrosarcoma (n=5).


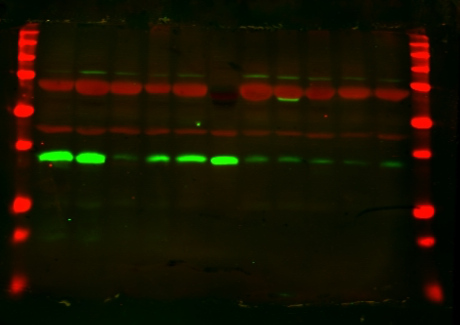


**CHORDOMA**

**CHONDROSARCOMA**

**Lasp1**

**ACTB**

***ACTB***

***(aspecific)***

**HeLa**

***100kDa***

***75kDa***

***37kDa***

***50kDa***

***25kDa***

**1**

**2**

**3**

**4**

**5**

**1**

**2**

**3**

**4**

**5**

**Fig. S1** Western blot of five skull-base chordoma, five chondrosarcoma and HeLa (control) samples showing LASP1 and ACTB protein expression at 36 and 42 kDa, respectively. ACTB also shows an aspecific signal in both chordoma and chondrosarcoma at approximately 70kDa.
